# In Vivo Production of HN Protein Increases the Protection Rates of a Minicircle DNA Vaccine against Genotype VII Newcastle Disease Virus

**DOI:** 10.3390/vaccines9070723

**Published:** 2021-07-02

**Authors:** Zhannan Wang, Xiaohan Zhao, Ying Wang, Chao Sun, Ming Sun, Xingyun Gao, Futing Jia, Chenxin Shan, Guilian Yang, Jianzhong Wang, Haibin Huang, Chunwei Shi, Wentao Yang, Aidong Qian, Chunfeng Wang, Yanlong Jiang

**Affiliations:** College of Animal Medicine, Jilin Provincial Key Laboratory of Animal Microecology and Healthy Breeding, Jilin Provincial Engineering Research Center of Animal Probiotics, Key Lab of Animal Production, Product Quality and Security, Ministry of Education, Jilin Agricultural University, Changchun 130118, China; wangzhannan2021@163.com (Z.W.); zzzxxxhhh2021@163.com (X.Z.); wangying202106@163.com (Y.W.); sunchao0602@163.com (C.S.); sunming202106@163.com (M.S.); gaoxingyun2021@163.com (X.G.); puffting@163.com (F.J.); scx1394706137@163.com (C.S.); yangguilian@jlau.edu.cn (G.Y.); jianzhongw@jlau.edu.cn (J.W.); nmdhuanghaihin@163.com (H.H.); shichunwei09@163.com (C.S.); yangwentao@jlau.edu.cn (W.Y.); qianaidong0115@163.com (A.Q.)

**Keywords:** minicircle DNA, CRIM, dual promoter, NDV vaccine

## Abstract

The Cre-recombinase mediated in vivo minicircle DNA vaccine platform (CRIM) provided a novel option to replace a traditional DNA vaccine. To further improve the immune response of our CRIM vaccine, we designed a dual promoter expression plasmid named pYL87 which could synthesize short HN protein under a prokaryotic in vivo promoter P_pagC_ and full length HN protein of genotype VII Newcastle disease virus (NDV) under the previous eukaryotic CMV promoter at the same time. Making use of the self-lysed *Salmonella* strain as a delivery vesicle, chickens immunized with the pYL87 construction showed an increased serum haemagglutination inhibition antibody response, as well as an increased cell proliferation level and cellular IL-4 and IL-18 cytokines, compared with the previous CRIM vector pYL47. After the virus challenge, the pYL87 vector could provide 80% protection compared to 50% protection against genotype VII NDV in pYL47 immunized chickens, indicating a promising dual promoter strategy used in vaccine design.

## 1. Introduction

Newcastle disease virus (NDV) has posed severe economic threats to poultry production worldwide since its first discovery in 1926. A number of genotypes, including type I, II, III, et al., have been circulated around the world for a long time. It is worth noting that the genotype VII of NDV has been considered to be the dominant strain in China during the last decade, which has resulted in significant economic loss for poultry production [1]. However, the traditional commercial NDV vaccine, such as the La Sota strain, belongs to genotype II instead of VII. The development of a novel NDV vaccine targeting genotype VII has received more and more attention in recent years.

In addition to traditional vaccines, such as inactivated virus and attenuated vaccines, a number of genetic engineering vaccines have recently been developed. The DNA vaccine, which is composed of a plasmid expressing desired antigens in an immunized host, which can stimulate both system immune response and cellular immune response at the same time, has drawn increased attention since it is easy to manipulate and produce compared to traditional vaccines. However, there are still some disadvantages regarding to the use of the DNA vaccine in field, including the relatively lower immune response level after intramuscular or intradermal injection, with or without electroporation by a gene gun. To overcome this problem, we have designed a novel minicircle DNA (mcDNA) vaccine platform previously named Cre-recombinase mediated in vivo mcDNA (CRIM), in which the complete plasmid could transform into mcDNA in vivo after immunization, yielding an increased humoral immune response and better protection against a wild-type NDV challenge [2]. However, we are still not satisfied with our novel CRIM system regarding to its relatively lower and slow humoral immune response, even though it is still higher than the traditional plasmid DNA vaccine, and we are trying to improve our construction further.

“DNA prime and protein boost” has been considered to be a classic approach for improving the immune response of DNA vaccines for a long time. On the other hand, there have been a few studies on immunizing both plasmid DNA and proteins at the same time [3,4], instead of the previous “DNA first followed by protein boost” strategy. Interestingly, immunization with both DNA and proteins of the S1 protein of SARS-Cov2 has also stimulated dramatically increased humoral immune responses compared with individual immunization with DNA or protein alone, eliciting full protection against the challenge of SARS-CoV-2 in a nonhuman primate (NHP) model. In particular, the codelivery of DNA and protein vaccines (DNA + Protein) was equally immunogenic to the sequential DNA prime-protein boost approach in eliciting specific IgG antibody responses and neutralization antibody responses [4]. The prime boost strategy has also been well studied in a chicken model. Junfeng Sun et al. [5]. described combined vaccinations with the plasmid DNA pCAG-optiF-2 prime + protein roptiF-2 boost vaccination strategy, eliciting more robust immunity, as confirmed by the detection of antibodies against NDV using enzyme-linked immunosorbent assay and virus neutralization assay, when compared to those vaccinated with only the plasmid pCAG-optiF-2 or protein roptiF-2, providing more efficacious protection against the virulent NDV challenge. Similar results were also observed regarding the H9N2 influenza virus [6], *Campylobacter jejuni* [7], and the H5N1 influenza virus [8]. However, there is still no report using plasmid DNA together with protein as immunogens in poultry. All the above-mentioned studies in NHP models drive us to illustrate whether it could be possible to immunize plasmid DNA with protein together in chicken, instead of using the traditional prime-boost strategy, for better immune response.

To stimulate a satisfied host immune response, one of the critical factors lies in the high expression levels of foreigner antigens. On the other hand, the high levels of antigen synthesis can also result in a metabolic burden to the vaccine strain, leading to a number of unwanted effects, including hyper attenuation, loss of viability, loss of plasmid, modified or poorly expressed antigen genes, and reduction in colonizing ability, ultimately resulting in poor immunogenicity [9]. To avoid these side effects, a series of in vivo induced promoters such as P_PagC_, P_nirB_, and P_ssaG_ have been employed, which could drive gene expression in cultured macrophages or in host tissues but poorly express in common laboratory media [10]. PagC is an outer membrane protein important for survival in macrophages [11], and the P_pagC_ promoter has been shown to function in different species. In studies comparing a number of promoters, P_pagC_ was found to have the greatest activity in murine tissues [12]; combined with its low in vitro activity, this has made it an attractive choice for driving antigen expression.

Regulated delayed lysis *Salmonella* is a novel designed attenuated *Salmonella* vector for vaccine study, dependent on the arabinose regulated *araC* P_BAD_ promoter and its regulation on the production of *asd* and *murA* genes, which are both necessary for the synthesis of bacterial cell walls [13,14]. The *Salmonella* strains can gradually lyse in vivo after oral immunization due to the absence of arabinose in vivo, yielding the release of produced protein antigens or plasmid DNA, resulting in an increased humoral immune response compared with a traditional attenuated *Salmonella* vectored vaccine.

In this study, we designed a novel CRIM vector based on regulated delayed lysis *Salmonella* which could synthesize HN protein under an in vivo regulated promoter P_PagC_, in addition to the previous mcDNA construction pYL47 [2], named pYL87. The novel designed CRIM vector could be used as an mcDNA vaccine as well as a protein subunit vaccine at the same time, which would provide us with an option to improve the immune response of the DNA vaccine.

## 2. Materials and Methods

### 2.1. Bacterial Strains and Growth Conditions

The bacterial strains and plasmids used in this study are listed as shown in Table 1. *Escherichia coli* (*E. coli*) χ6212 cells were used for plasmid construction, and *Salmonella* strain χ11218 was used as an immunization host strain. Luria-Bertani medium (LB) supplemented with 50 μg/mL diaminopimelic acid (DAP) (Sigma, Livonia, MI, USA) was used for bacteria culture at 37 °C with shaking, and 0.1% arabinose (Sigma, Livonia, MI, USA) was included when necessary. MgM medium [15] was used for the induction of P_pagC_ promoter when necessary, consisting of 100 mM Tris-Cl, pH 5.0 or 7.4, 5 mM KCl, 7.5 mM (NH_4_)_2_SO_4_, 0.5 mM K_2_SO_4_, 1 mM KH_2_PO_4_, 8 mM MgCl_2_, 38 mM glycerol, and 0.1% casamino acids.

### 2.2. Plasmids Construction

The primers used in this study are listed in Table 2. A fragment of 1495 bp was amplified using primers HNopt1-NcoI-F/HNopt1-HindIII-Rfrom plasmid pUC-HN as template and inserted into plasmid pYA3342-pagC, yielding pYL66 which could synthesize a short HN protein under P_pagC_ promoter when induced in MgM medium. Then, primers PagC-SacII-F/TT-SacII-R were used to amplify the PpagC-HNopt-TT fragment and inserted into plasmid pYL47 at the SacII site, resulting in the dual promoter construction pYL87, which would produce HN proteins under both CMV promoter and P_pagC_ promoter. On the other hand, pYL46, which harbors EGFP reporter gene under the control of CMV promoter, was used to insert the same PpagC-HNopt-TT fragment at SacII site, yielding pYL86, which was designed for comparative purposes.

### 2.3. Determination of Synthesized HN Proteins from Both Prokaryotic and Eukaryotic Promoters

*Salmonella* strain χ11218 harboring individual plasmids was incubated overnight at 37 °C with shaking in LB medium with 0.2% arabinose. The bacteria were then inoculated into fresh LB medium with 0.2% arabinose at a dilution of 1:100 and cultured further until OD_600_ achieved about 0.8. After that, 1 mL of individual strain was collected by centrifuge and washed three times with MgM medium. Then, 1 mL MgM medium with 0.2% arabinose was used to resuspend the pellet and incubate for another 4 h. Then, the bacteria were collected by centrifuge and resuspended with SDS loading buffer for further Western blot assay using HRP-labeled mouse anti-FLAG antibody (1:10,000, Sigma, Livonia, MI, USA).

The plasmids were then purified with the GoldHi EndoFree Plasmid Midi Kit (Kangwei Co., Beijing, China) and transfected into 293T cells using Lipofectamine 3000 (Life Technologies, Carlsbad, USA) according to the manufacturer’s instructions. In detail, 1 × 10^5^ cells per well in 24-well plates were transfected with 1 μg individual plasmid. After 48 h incubation, the cells were determined by the immunofluorescence assays (IFA) as described previously [16] using rabbit anti-Cre antibody (1:500, GeneTex) and mouse anti-His monoclonal antibody (1:1500, Abbkine) as the primary antibody, respectively, followed by Cy3-labeled goat anti-rabbit IgG (H + L) (1:500, Beyotime, China) and FITC-labeled goat anti-mouse IgG antibody (1:500, Cwbiotech, Beijing, China), as the secondary antibody. Fluorescence was observed under a confocal microscope (LSM710, Zeiss, Oberkochen, Germany).

### 2.4. Determination of the Production of mcDNA in Transfected Cells

The nuclear DNA was extracted and subjected to PCR amplification to confirm the presence of mcDNA. In detail, 1 × 10^6^ 293T cells per well in 6-well plates were transfected with 2 μg individual plasmid. After 48 h incubation, the cells were collected, and the nuclear was extracted by using a nuclear extraction kit (BestBio, Shanghai, China) followed by DNA extraction using SDS containing lysis buffer [17] and PCR amplification using primers CMV-F1/CMV-R1. In addition to pYL47, pYL86, and pYL87 constructions, the empty vector pYA4545 was also included as control. The original pYL47 plasmid without transfection process was also included as template for PCR analysis.

### 2.5. Chicken Immunization and Samples Collection

Chicken experiments were performed using laying hens (30 days old) (Hongda Animal Technology Co., Ltd., Changchun, China). In detail, 1-day-old laying hens were fed about 30 days until the parental hemagglutination inhibition (HI) antibody titers dropped under 4log_2_ and then divided into six groups, with 16 chicks per group. Approximately 1 × 10^9^ CFU of χ11218 strains harboring individual pYA4545, pYL47, pYL86, and pYL87 were used for oral immunization at day 30 (recorded as day 0 post vaccinations, 0 dpv). Then, boost immunizations with the same dose were performed at a two-week interval at 14 dpv, 28 dpv, and 42 dpv, respectively. Chickens inoculated with BSG buffer were also included as negative controls. In addition, the commercial genotype VII NDV vaccine (Jiangsu Nannong Hi-Tech Co. ltd., Nanjing, China) was also used to immunize chicks at 0 dpv by intramuscular immunization as positive control. The serum samples were collected at 21 dpv, 35 dpv, and 49 dpv to determine the HI antibody levels using 1% chicken red blood cells (RBCs) with 4 hemagglutination (HA) units of the NDV-specific antigen (NA-1, Jilin University, Changchun, China).

### 2.6. Cell Proliferation Assay

The splenocytes from vaccinated birds were prepared as described previously [18] with minor changes. Briefly, the spleen samples were collected at 49 dpv (*n* = 3), and single cell suspensions were prepared by cutting the spleens into small pieces, gently dissociating the spleen pieces on a sterile copper wire mesh, and then purified by gradient centrifugation (500× *g*, 30 min) using Histopaque 1077 (Sigma, St. Louis, MO, USA). After washing with phosphate-buffered saline (PBS) three times, splenocytes were resuspended in RPMI 1640 medium (Hyclone, South Logan, UT, USA). Then, 100 µL (10^6^ cells/mL) was added to each well in a 96-well plate, incubating with 10µg/mL purified HN protein with triplicate repeats at 37 °C and 5% CO_2_ for 48 h. After incubation of cells in counting kit-8 (CCK-8) solution (Beyotime Biotechnology, Shanghai, China) (10µL) for a further 6 h, cells were evaluated using OD_490_ value measured by spectrophotometer (Bio-Rad, USA).

In addition, the supernatants from cells incubated with HN protein for 72 h mentioned above were also collected and the production of IL-4, IFN-g, IFN-a, and IL-18 was measured by commercial kits following the manufacturer’s instructions (Kete Inc., Jiangsu, China).

### 2.7. Challenge Study

The genotype VII NDV wild-type strain NA-1 (kindly provided by Pro Zhuang Ding and Yanlong Cong from Jilin University) was used to challenge at 56 dpv at a dose of 10^6^ ELD_50_ via nasal drops (*n* = 13). During the next 10 days, the clinical signs and survival rates were recorded. At day 5 post challenge, three birds were sacrificed from each group, and the lung samples were collected and subjected to viral titer determination. In detail, the lung samples were collected, homogenized in cell culture medium (0.1 g/mL), and clarified by centrifugation. The viral titers in the tissue samples were then determined by limiting dilution on chicken DF-1 cells.

### 2.8. Statistic Analysis

The data were analyzed using GraphPad Prism 8.3 software. Data are presented as the means ± standard error of the mean (S.E.M.) and analyzed by one-way ANOVA (Dunnett’s multiple comparison test) of at least three independent experiments. Values of *p* < 0.05 were considered to indicate statistically significant differences.

## 3. Results

### 3.1. Construction of Plasmid Expressing HN Protein with Both Prokaryotic and Eukaryotic Promoters

**Figure 1:**
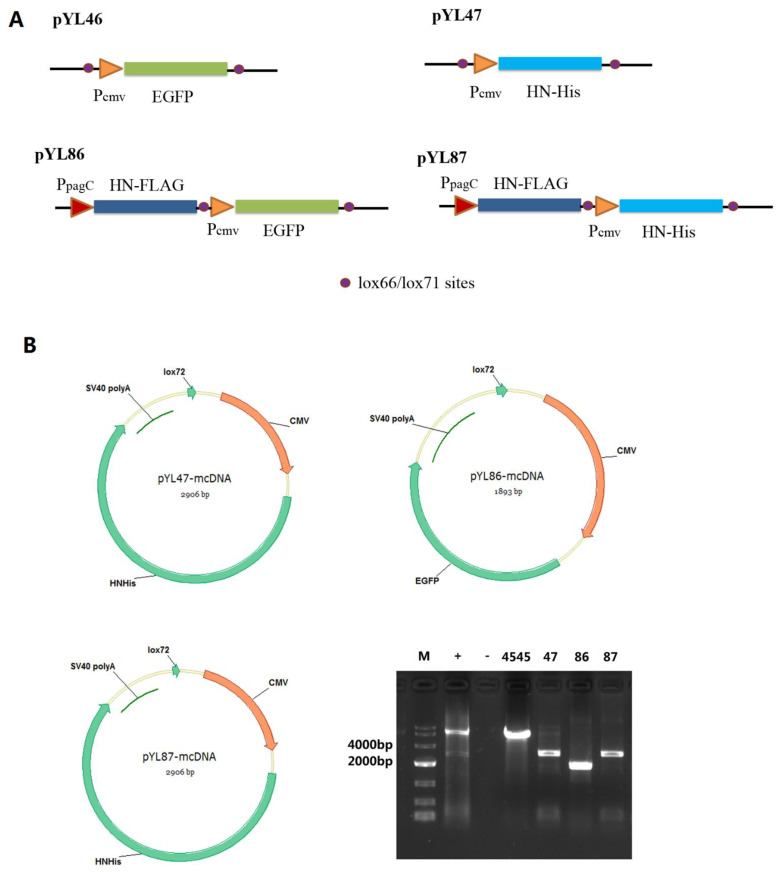
Construction of designed plasmids. pYL46 and pYL47 were used as original templates by inserting P_pagC_-HN-TT cassette, yielding pYL86 and pYL87, respectively (**A**). The minicircle DNA derived from pYL47, pYL86, and pYL87 was confirmed by DNA extraction from cells transfected with individual plasmids, followed by PCR amplification (**B**). M: DNA marker DL10000 (Takara); +: PCR amplification using parental pYL47 plasmid as positive control; -: PCR amplification without template as negative control; 4545: PCR amplification using the extracted DNA from pYA4545 transfected cells as template; 47,86,87: PCR amplification using the extracted nuclear DNA from individual plasmid transfected cells as template.

In our previous study [2], we designed an in vivo mcDNA platform for veterinary vaccine application, named CRIM. In this novel construction, the Cre-recombinase was driven by the strictly eukaryotic promoter Ppgk, which showed almost no activity in an in vitro condition. After transfection, the Cre-recombinase expressed by Ppgk promoter could mediate recombination between the two loxP sites located outside the antigen synthesis cassette, resulting in the formation of mcDNA spontaneously. We constructed two plasmids expressing EGFP or HN protein of the genotype VII NDV under the CMV promoter, named pYL46 and pYL47, respectively (Figure 1A). The prokaryotic expression cassette PpagC-HNopt-TT was amplified and inserted into the SacII site of both plasmids, yielding pYL86 and pYL87, respectively (Figure 1A). The HNopt was designed to exclude the anchoring sequence of HN protein for better prokaryotic synthesis. To confirm the production of mcDNA in cells after transfection, the nuclear DNA were extracted and used as a template for PCR amplification using primers. As expected, all the CRIM designed plasmids (pYL47, pYL86, and pYL87) could transform to mcDNA by themselves after transfection, whereas the control plasmid pYA4545 (6800 bp, without Cre-recombinase and loxP sites) still remained in the original conformation, as opposed to that of mcDNA (Figure 1B). It is worth noting that both plasmids pYL47 and pYL87 harbored the CMV-HN-polyA cassette, whereas the pYL86 harbored the CMV-EGFP-polyA cassette instead, yielding larger PCR products in both groups compared with pYL86 (2906 bp vs. 1893 bp).

### 3.2. Confirmation of Desired HN Protein Synthesis from Both Prokaryotic and Eukaryotic Promoters

To confirm the production of HN proteins from both promoters, immunofluorescent assay and Western blot were performed as described above. The P_pagC_ promoter was an in vivo regulated promoter which was under the control of the lysosome acid environment, usually used as a regulated promoter in vaccine studies. The P_pagC_ promoter could also be induced in vitro by MgM medium [9], which stimulates the function of the P_pagC_ promoter by acid culture condition, similar to the environment encountered after *Salmonella* translocates into macrophage cells.

**Figure 2:**
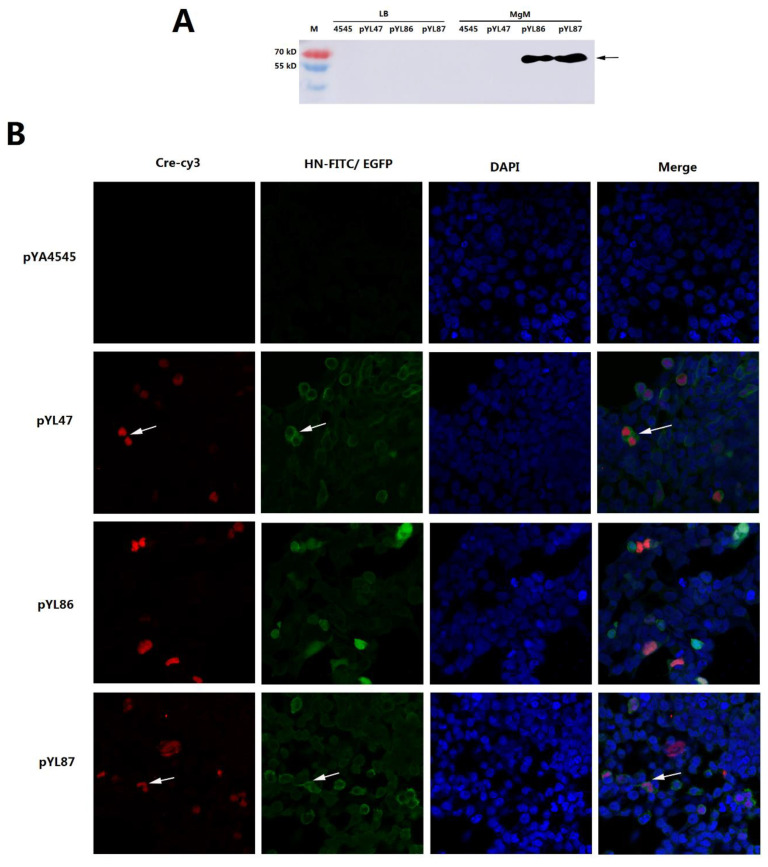
Determination of desired antigen synthesis by Western blotting and immunofluorescent assay (IFA). The prokaryotic promoter P_pagC_ synthesized HN proteins in MgM medium in pYL86 and pYL87 (**A**). M: prestained protein marker; 4545, pYL47, pYL86, and pYL87: *Salmonella* strain χ11218 harboring individual plasmids cultured in LB medium and MgM medium, respectively. The eukaryotic promoter CMV produced HN proteins that were confirmed by IFA (green), while the production of Cre recombinase was also confirmed (red)(**B**).

To identify the different expression of HN from prokaryotic or eukaryotic promoters, His-tag and FLAG-tag were fused with different HN proteins under eukaryotic and prokaryotic promoters, respectively. After induction in MgM medium for 4 h, the production of HNopt under the P_pagC_ promoter was confirmed in both pYL86 and pYL87 plasmids, while no obvious protein synthesis was observed when cultured in LB medium (Figure 2A), indicating that the P_pagC_ promoter could work as an in vivo regulated promoter. The plasmid pYL47 without the PpagC-HNopt-TT cassette was also included as a negative control. The production of HN protein from eukaryotic promoter in both pYL47 and pYL87 was observed by IFA as described with a green color (Figure 2B). It is worth noting that since the pYL86 could synthesize the EGFP protein under the CMV promoter, it also appeared to be a green color. On the other hand, the presence of Cre recombinase in transfected cells was also confirmed in all the constructions, as shown in red.

### 3.3. Increased HI Antibody Titers Induced by Double Promoter Design

**Figure 3:**
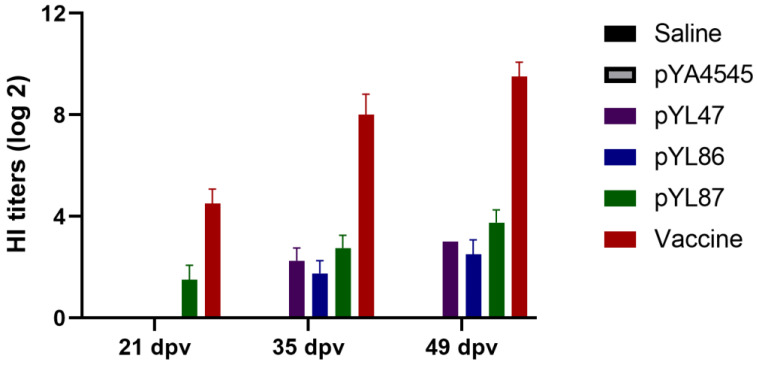
The haemagglutination inhibition (HI) antibody titers were determined at days 21, 35, and 49 postvaccination (dpv).The results are shown as means ± SEM (*n* = 3).

The HI antibody titers were determined to evaluate whether the production of HN protein driven by the P_pagC_ promoter could enhanced the humoral immune response in chickens. Since we observed obvious pre-existing parental HI antibodies, we had to wait until 30 days to exclude the possible interference effects of vertical transmission HI antibodies on vaccination. As expected, the chickens in the pYL87 immunized group began to show HI positive results on day 21 postvaccination, even though the average titers were only about 1.5, whereas no detectable HI antibody was observed in both the pYL47 and pYL86 immunized chicks (Figure 3). As time elapsed, the HI antibody titers in the pYL47 and pYL86 groups were also detectable at 35 dpv, with an average value of 2.25 and 1.75, respectively. At the same time, the HI titers in the pYL87 group achieved about 2.75 on average. At 49 dpv, all the HI titers increased compared with 35 dpv, with average values of 3.0, 2.5, and 3.75, respectively. On the other hand, the HI titers in the inactive vaccine group increased from 4.5 to 8 and 9.5 at 21 dpv, 35 dpv, and 49 dpv, respectively. It is worth noting that the eukaryotic CMV promoter alone (pYL47) seemed to result in slightly better HI titers compared to the prokaryotic P_pagC_ promoter alone (pYL86), even without statistical differences. Unfortunately, although we noticed increased HI titers in the dual promoter (pYL87) group compared with pYL47, statistically significant effects of the prokaryotic P_pagC_ promoter on HI titers were still not observed. On the other hand, the commercial inactivated vaccine induced the highest HI antibody titers as expected, indicating that there is still much room for improvement with our novel CRIM vaccine.

### 3.4. Cell Proliferation

**Figure 4:**
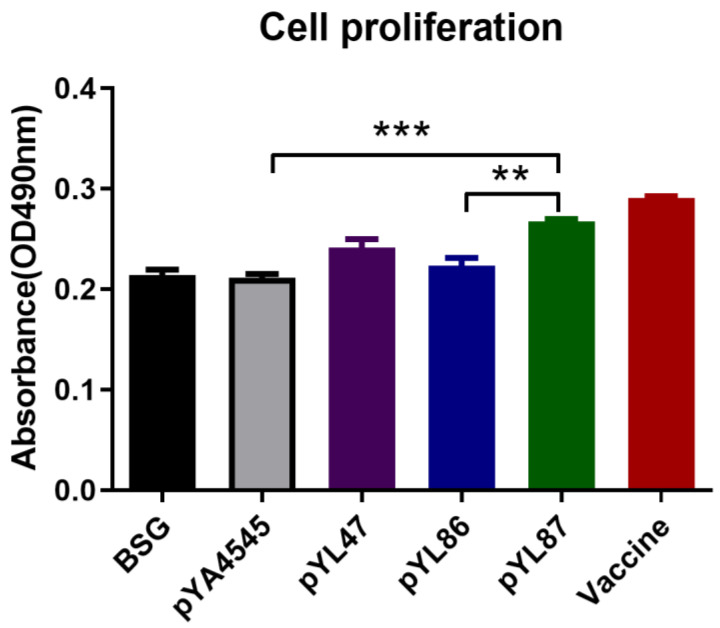
Cell proliferation assay. The spleen cells were separated and stimulated with purified HN protein, followed by observation with CCK-8 assay. The results are indicated as means ± SEM (*n* = 3). The statistical significance was calculated by one-way ANOVA and a Tukey post-test. ** *p* < 0.01; *** *p* < 0.001.

The CCK-8 bioassay was performed to determine the proliferation of splenocytes in the presence of purified HN protein. The results showed that immunization with the dual promoter construction pYL87 significantly increased its proliferation compared with pYL86 (*p* < 0.01) and the empty vector pYA4545 (*p* < 0.001). A similar trend was also observed regarding pYL87 vs. pYL47, even though no statistical difference was observed (Figure 4).

### 3.5. Cytokine Production

**Figure 5:**
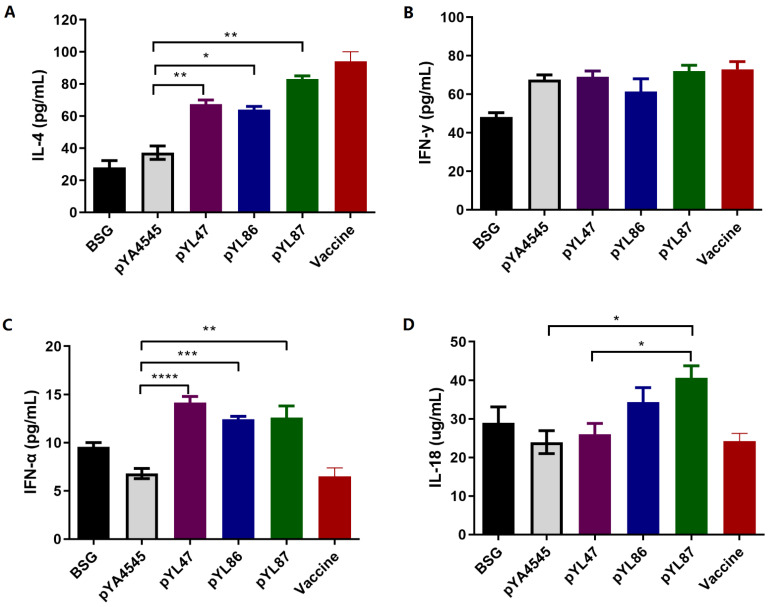
Production of IL-4, IFN-g, IL-18, and IFN-a cell supernatants stimulated with purified HN protein. The concentrations of IL-4 (**A**), IFN-g (**B**), IFN-a (**C**), and IL-18 (**D**) were determined by ELISA assay. The results are indicated as means ± SEM (*n* = 3). The statistical significance was calculated by one-way ANOVA and a Tukey post-test. * *p* < 0.05; ** *p* < 0.01; *** *p* < 0.001; **** *p* < 0.0001.

The concentrations of IL-4, IFN-g, IFN-a, and IL-18 in collected supernatants of spleen cells incubated with HN protein were determined by ELISA kits. Similar to the previous report [2], the production of IL-4 was more obvious compared with IFN-g, in which the immunization with pYL47, pYL86, and pYL87 stimulated an increased yield of IL-4 compared with the empty vector pYA4545 (Figure 5A), whereas no significant production of IFN-g was observed among all the groups (Figure 5B). The concentrations of IFN-a and IL-18 were also measured. Interestingly, all immunized birds with pYL47, pYL86, and pYL87 strains demonstrated increased production of IFN-a compared with BSG and the empty vector, even with the inactivated vaccine group (Figure 5C). On the other hand, the immunization with pYL87 stimulated significant production of IL-18 compared with both pYA4545 and pYL47 (Figure 5D).

### 3.6. Dual Promoters Construction Increased the Survival Rate and Decreased the Viral Load

**Figure 6:**
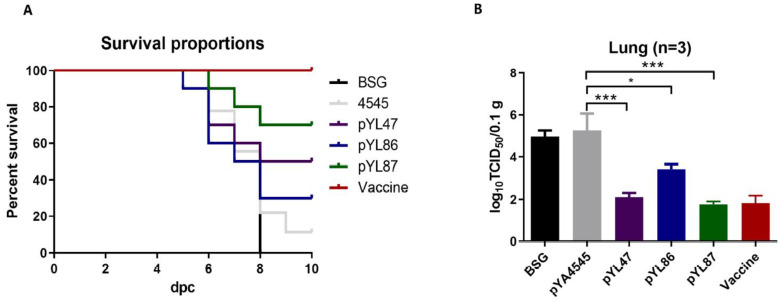
Protection against virulent NDV challenge. The chickens were challenged with 10^6^ ELD_50_ NDV NA-1 strain and observed for 10 days, and the survival curves were calculated (**A**). In addition, lung samples were also collected at day 5 post challenge, and the viral titers were determined in DF-1 cells (**B**). * *p* < 0.05; *** *p* < 0.001.

Two weeks after the last immunization (56 dpv), wild-type VII NDV was used to challenge as described above, and chickens were observed for the next 10 days. Beginning on day 3 post challenge, the chickens in the BSG and pYA4545 groups showed various degrees of clinical signs, such as cough and depression, whereas fewer chickens began to be infected at day 5 to 6 post challenge in the *Salmonella* immunized chickens. The chickens began to die at day 5 post challenge in the BSG and pYL86 groups, whereas the other *Salmonella* immunized chickens began to die at day 6 (Figure 6A). At the end of the study, compared with the BSG and empty vector groups in which 100% (8 days post challenge) and 90% mortality rates were observed, respectively, immunization with strains harboring pYL86 and pYL47 induced a survival rate of 30% and 50%, respectively. Consistent with the results of HI titers and cell proliferation assay, immunization with the dual promoters pYL87 strain resulted in a 70% survival rate, indicating its increased protection against NDV challenge compared with the single promoter construction. On the other hand, no obvious clinical signs could be observed in the inactivated vaccine group during the whole challenge process, and a 100% survival rate was observed.

To further confirm the protective effects, the viral titers in lung samples were evaluated using DF-1 cells. The results showed that the highest titers were observed in both the BSG and empty vector groups, whereas all the other *Salmonella* immunized chickens as well as the inactivated vaccine group showed significantly lower titers (Figure 6B). It is worth noting that the virus titers in the pYL86 immunized chickens appeared to be slightly higher compared with pYL47 and pYL87, as well as the inactivated vaccine, which is consistent with the immune responses mentioned above.

## 4. Discussions

The “prime-boost” immunization process has been considered to be an effective strategy to enhance the immunogenic prosperities of the DNA vaccine. On the other hand, it has been noticed recently that the combination of both plasmid DNA and purified proteins induced a significantly enhanced humoral immune response compared with either DNA/protein alone or separate immunization at different sites. For example, the macaques in the coadministration group developed higher Env-specific humoral and cellular immune responses, suggesting that the simultaneous recognition, processing, and presentation of DNA + Env protein in the same draining lymph nodes play a critical role in the development of protective immunity [19]. Inspired by these interesting findings, we meant to design an automate mixture of both plasmid DNA and synthesized protein antigen, delivered by a self lysis attenuated *Salmonella* vector. In addition, the in vivo promoter P_pagC_ was employed to synthesize HN protein in our study. The regulated P_pagC_ promoter theoretically only produced HN protein once the *Salmonella* colonized in the host cells, which could help to alleviate the metabolism burden to the *Salmonella* strain in vitro. Therefore, ideally, once the lysis *Salmonella* enter into the host cell, the acid atmosphere in the lysosome could stimulate the activity of the P_pagC_ promoter, while the plasmids released by *Salmonella* lysis could also be used as a DNA vaccine.

Different from a traditional plasmid DNA vaccine, we included our novel CRIM vector in the present study. The CRIM vector can turn into mcDNA by itself once the plasmid delivered by the *Salmonella* is released into the host cell cytoplasm, resulting in a more enhanced adaptive immune response compared with the traditional DNA vaccine. The combination of mcDNA technology and an in vivo promoter could theoretically induce a better immune response. As expected, the pYL87 construction with the novel design could somehow stimulate increased HI antibody titers compared with both pYL47 and pYL86, which harbored either mcDNA or the P_pagC_-HN cassette alone (Figure 3). However, the HI titers in the pYL87 immunized chickens did not seem to be statistically different from either pYL47 or pYL86 alone. One of the possible explanations for these disappointing results was that the selected HN gene fragment under the P_pagC_ promoter lacked any secretion signal sequences. The prokaryotic expressed HN protein thus could only be located in *Salmonella* strains, without any secretion during replication. As a result, the prokaryotic produced HN proteins were only released into cells which were invaded by *Salmonella* and probably not belonging to any antigen presenting cells, resulting in only slightly increased HI titers, far from our original expectation. On the other hand, the inactivated vaccine could induce high levels of HI antibody titers and provide 100% protection, indicating that other strategies could be considered to further improve our current vaccine candidates, such as the DCs targeting approach by a single chain variable fragment or DCs targeting peptides.

Similar results were also observed regarding the cell proliferation assay (Figure 4), indicating the presence of prokaryotic HN protein and indeed achieving our original hypothesis. A previous study showed the rapid and robust development of SIV Env antibody responses in Rhesus Macaques when protein and DNA were coadministered in the same muscle in the priming vaccination compared to a separate administration group. In addition, measurements of cellular immunity at the same time point in peripheral mononuclear cells showed significantly higher levels of Env-specific T cell responses in the coadministration group [19]. Although there must be a number of differences between mammals and chickens with regard to the host immune system, immunization with both protein and DNA at the same site could be one of the possible explanations for increased HI titers and cell proliferation observed in our study.

We also determined the antigen-specific IL-4 and IFN-g in the supernatant of the spleen cells incubated with purified HN protein. An increased production of IL-4 was observed in the pYL87 group compared with both pYL47 and pYL86, whereas there were no obvious differences regarding IFN-g, indicating that the dual promoters mediating antigen synthesis biased the humoral immune response to the Th2 type instead of the Th1 subtype. We also found some interesting cytokine production profiles, such as IFN-a and IL-18. The type I interferon IFN-a has been shown to augment the cellular immune response to DNA vaccination against HCV core protein [20]. The increased production of IFN-a in the pYL47, pYL86, and pYL87 immunized chickens possibly provides another explanation for the observed protection against virus challenge in addition to humoral immune response. Another interesting finding was the increased production of IL-18, which has been widely used as an efficient vaccine adjuvant [21,22]. The presence of both prokaryotic and eukaryotic production of HN protein in the pYL87 group significantly increased the concentration of IL-18, indicating that IL-18 could be another possible reason for the enhanced protection against virus challenge, compared with the original eukaryotic expressing vector pYL47.

Although we have achieved our original plan to improve the humoral and cellular immune response regarding our previous CRIM vector, pYL47, we are still not satisfied with the current pYL87 construction version. The average HI titers after four times of immunization are only around 4log_2_, which is much lower compared to the inactivated vaccine control, indicating that there is still much progress to be made in order to achieve our final goal. In conclusion, the novel dual promoter synthesized HN protein provides us with a novel option to enhance the efficiency of traditional DNA vaccines instead of the traditional “prime-boost” strategy.

## Figures and Tables

**Table 1:** Bacterial strains and plasmids used in this study.

Plasmids or Strains	Description	Source
Strains		
χ11218	*Salmonella* host strain for DNA vaccine study	Roy Curtiss III University of Florida
χ6212	*E.coli* host strain for DNA cloning	Roy Curtiss III University of Florida
Plasmids		
pYA4545	Eukaryotic expression vector in *Salmonella*, arabinose dependent	Roy Curtiss III University of Florida
pYA3342-pagC	Asd^+^ plasmid, P_pagC_ promoter	Lab collection
pYL66	Asd^+^ plasmid, express HN under P_pagC_ promoter	This study
pUC-HN	Kan^+^, complete HN gene of genotype VII NDV cloned into pUC57 vector	Lab collection
pYL46	Minicircle DNA vector express EGFP under CMV promoter	Lab collection
pYL47	Minicircle DNA vector express HN under CMV promoter	Lab collection
pYL86	Insert PpagC-HN-TT cassette into pYL46	This study
pYL87	Insert PpagC-HN-TT cassette into pYL47	This study

**Table 2:** Primers used in this study.

Primers	Sequences
HNopt1-NcoI-F	TGccatggGACCGCACGACCTCGCAGGTA
HNopt1-HindIII-R	GATaagcttCTATTACTTGTCGTCGTCGTCCTTGTAGTC
PagC-SacII-F	TCCccgcggGTTAACCACTCTTAATAAT
TT-SacII-R	TCCccgcggAAGAGTTTGTAGAAACGCAA
CMV-F1	GTACGCCCCCTATTGACG
CMV-R1	TTGGCATATGATACACTTG

## Data Availability

Not applicable.
